# Ninth Annual Meeting of the American Society of Dental Surgeons, Held at Saratoga, August, 1848

**Published:** 1851-04

**Authors:** William Henry Burr

**Affiliations:** Phonographer


### NINTH ANNUAL MEETING OF THE AMERICAN SOCIETY OF DENTAL SURGEONS, HELD AT SARATOGA, AUGUST, 1848.

Reported by WILLIAM HENRY BURR, Phonographer.

*Continued from January Number.*

My wedges are generally made of pine wood, but I have used hickory, cedar, dog-wood and India rubber with equal advantage, and in numerous cases, I have not used anything with greater satisfaction than cotton, it possesses the double value of pressing aside the gum which sometimes partly covers the cavity and it separates the teeth at the same time, thus enabling the operator to fill without annoyance from bleeding. The file may be used with benefit, and more freely upon the bicuspids and molars, than upon the incisor teeth; when well filed, so as to be kept apart, they retain their stopping better than when their sides approximate so as to touch each other.

I have within the last few weeks wedged the teeth apart for the purpose of examining them, of the lady who came to me at the age of 12, before alluded to, and found them as perfect and free from decay, as on the day they were stopped, now about 16 years ago.

As regards filing, I have had no reason to change my opinion of its importance, since the publication of my first little Brochuse, in 1820, according to the facts of my experience. I then regarded the file in the hands of the skilful operator a most valuable instrument, none more so, but it should be used with great caution, otherwise it is the most destructive of all implements, and has ruined more teeth than all others put together. I had a case of a child, probably about 12, the enamel of whose teeth was of that soft perishable character, that it seemed almost impossible to do anything to save them. They could not be stopped, their sides being wholly decayed, but the decay had not as yet penetrated to any considerable depth. In order to get sufficient room between the teeth to enable her to clean them, I cut a large portion from the side of each tooth with a common sized round file, giving the teeth a wedge-like shape, the neck representing the thickest part of the wedge, and polishing the filed surfaces as well as I could. At that time, I did not believe that these teeth could last beyond a very few years, but ten or twelve years have now elapsed, and they remain perfectly good, and will probably continue so during life.

In moving teeth by wedges, I do not imagine that the point of the fang is stirred, nor have I ever known a nerve ruptured or injured by careful wedging.

It is now about eighteen years since Mr. J. Parmly made a little machine, not unlike a miniature windlass in form and power, for the purpose of bringing a superior cuspidatus into its place, which fell completely within or behind the lower cuspidatus and first bicuspis. The operation of moving it occupied about three weeks, it was a large and firm tooth which had taken that direction from the temporary cuspidati not having been removed in time, which retained its place covering or hiding from view the one in question until the lady was about twenty-eight years old, when it loosened and dropped out, leaving the very unsightly defect which we were then called upon to remedy. When the lady was about twenty, I proposed performing the operation, which at that time was declined as a thing impracticable, herself and friends having been previously told that "it could not be done." The tooth in question, was moved a greater distance, and at a later period of life than any one I have ever known, yet it remains firm in its socket to this day, while some of its neighbors have been lost from alveolar absorption.

It is necessary to caution our patients, as to the question of wedging, many are apt to suppose from seeing it done, that it is a very small and easy matter; and I have had persons come to me with large pieces of wood forced between their teeth, thus opening up a gap to almost the width of an entire tooth. I have always cautioned such, not to use wedges without definite and proper directions, and I would recommend all others to do so.

Dr. Westcott here showed one of his first growth of teeth, which remained still, and asked Dr. Parmly's opinion whether it should be extracted.

The chairman said, if he was a patient of twelve years of age he would have it taken out.

Dr. Harris mentioned another case in point. A patient twenty-two years of age had a cuspid tooth extracted; some six weeks after he applied to Dr. H., for an artificial tooth, and it was set for him. Six months afterwards, a permanent cuspid made its appearance about a quarter of an inch behind the dental arch, but he succeeded ultimately in bringing it forward to its proper place.

Dr. Dunning wished to know Dr. Harris' experience with regard to the success of filling over exposed nerves—what was the advantage of the process of arching the filling over the nerve, to simply covering it with a cap.

Dr. Harris—If it had occurred to me previous to leaving home, I could have brought some statistics upon the subject, which might be interesting to the society. Wishing to obtain all the information I can concerning any new operation, I have been in the habit of noting not only the number of operations of every kind, but the circumstances under which they were performed. In 1833 I performed for the first time the operation of filing over an exposed nerve. The teeth were two central incisors, and being unwilling to destroy their nerves, as I believed that by so doing the chances of their permanent preservation would be lessened, and as I had frequently failed of success in capping the nerve I determined to fill without doing it. I did so, and the teeth remained healthy and sound for nearly seven years. I did not, however, repeat the experiment again for several years, deeming it a somewhat hazardous experiment. At length, however, I performed the operation again but this time it proved a failure, inflammation supervened, which ultimately terminated in the suppuration of the lining membrane. Some years after I repeated the operation and was successful, and until August, 1846, I had performed it, perhaps about twenty times, and fourteen with success. Since that time I have been more successful and during the past year, I have had to extract only three teeth treated in this manner.

The success of the operation not only depends upon the care with which it is performed, but also upon the absence of pain and inflammation at the time.

Dr. Westcott inquired if Dr. Harris filled teeth in this way, that came to him aching?

Dr. Harris.—Never. I never perform the operation when the lining membrane is inflamed. In such cases, if the preservation of the tooth is of great importance, I remove the entire contents of the tooth, either with an instrument immediately, or first destroy the vitality of the pulp with arsenic and afterwards remove it, and fill the entire pulp cavity. Capping the nerve when it can be properly done, is quite as good as my method, but the amount of skill required is such, that I do not succeed as often in this way as the other.

Dr. Asa Hill stated that he had a tooth capped about a year ago, by Dr. Foster, and it had been preserved as well as any tooth in his jaws.

Dr. Rich inquired what means Dr. Harris took to keep the cap in its position?

Dr. Harris replied, that he cut a groove or bearing for the cap in the walls of the cavity, then fitted it in such a manner as to prevent it from being easily displaced or moved by the introduction of the foil. But for the last three or four years I have not found it necessary to apply a cap previously to the introduction of the gold in a single instance.

Dr. Rich inquired if a cap of polished gold plate could be so placed that it would not move during the filling of the cavity if it would not be a better surface to be presented to the exposed nerve, than the rough one, that is presented where the arch (or cap) is formed by the filling, in the manner you have described?

Dr. Harris.—It is possible it might, yet in most cases the cap occupies so much room, that as a general rule, a filling cannot afterwards be as substantially introduced. Dr. Koecker was the first to recommend capping but he employed sheet lead; from the supposition that it possessed anti-inflammatory properties. I believe, however, that Dr. Fitch was the first to apply caps of gold.

The chairman here mentioned that his brother had used caps before Dr. Fitch published his work.

Dr. Rich.—I frequently fill teeth where the nerves are exposed. In such cases I use a gold cap to protect the nerve from pressure. I never find it difficult to place and retain the cap where I want it.

I will describe the manner in which the cap is made and placed over the nerve. I make a convex cap of polished gold plate, of such size and shape that it will rest upon the solid bone which surrounds the orifice opening into the cavity of the nerve. I fit this accurately, and polish and burnish the edge. For placing the cap over the orifice, I use a small instrument of steel wire, eight inches long; about two inches of this is drawn or filed down, to form a shank about the size of a number six sewing needle, and is left untempered to allow of its being bent. The point is bent round, so as to form a small ring or eye, of such size that an ordinary sized knitting needle will pass through it; the shank is then bent close to the eye, so that the eye will stand at right angle with that part of the instrument which forms the handle. Where it is necessary to place the cap in the required position, I bend the shank of the instrument just described to the proper form, and dip the eye in melted wax, and while the wax is warm, attach the instrument to the convex face of the cap, which is then placed in the proper position, where it is held firmly and gold foil packed around the edge of the cap until the cap is secure. The small instrument is then detached from it, and the wax carefully removed. At this stage of the operation, the cap presents the appearance of a jewel in its setting, and in fact is secured in the same way. After the wax is removed, gold foil is packed in and condensed until it is level with the apex of the cap. This generally occupies from one-third to one-half of the depth of the cavity in the crown. The gold foil thus introduced is then levelled and burnished, and forms an even solid floor to the main cavity, which is then filled up the same as any ordinary cavity.

By the method just described, the gold with which the cap is secured in its place, or the setting of the cap as it may be called, is entirely distinct and separate from the top layer with which the rest of the cavity is filled, so that in case any part of the top layer should be displaced, from accident or design, it would not interfere with the cap. I never attempt to treat a nerve in this way, unless I expose it myself. Slight hemorrhage sometimes follows the exposure; yet, in my practice, the operation rarely produces inflammation. I have often exposed the lining membrane without producing hemorrhage. With a good light and a steady and delicate hand, the membrane can be exposed five times out of six without hemorrhage.

Dr. Harris wished to inquire, in this connection, about the effect of cauterizing the nerve with a hot iron, and then filling. For his own part although highly recommended by Dr. Koecker, he was disposed to believe that it would be followed by inflammation and suppuration. The effort of nature to restore the injury, he thought, would produce this state of things.

Dr. Rich said that in cases where he had tried it, it produced that effect five times out of six. He wished to inquire if there were not others who had exposed the nerve without hemorrhage.

Dr. Dunning said that he had seen nerves exposed by the excision of roots, preparatory to the incision of pivot teeth, and afterwards filed up by the stump file, without hemorrhage.

Dr. Harris said, where vessels were ruptured by violence, they generally contract so as to prevent hemorrhage; whereas, if severed with a sharp instrument, they generally bleed. He had known one or two cases where there was no perceptible discharge of red blood.

Dr. Foster did not conceive it to be important, in making a cap to cover an exposed nerve, that the edges should be made round and smooth, but rather that they should be left rough and uneven, and so adapted to the parts of the cavity upon which they were to rest, particular care being taken that the size and form are in due proportion, as to secure it in its proper position. He had been successful, in a large majority of cases, in preserving the tooth alive. Whenever the death of the nerve ensues, he attributes it more to irritation from heat and cold, than from pressure; he had come to this conclusion from the fact that, in greatly increased proportion, he had lately been successful in treating these cases, owing, as he believes, to this precaution, viz. placing some substance which is a non-conductor, between the exposed nerve and the concave surface of the gold cap. For this purpose he had used some of Dr. Hill's composition, warming it, and compressing a very small portion with a convex instrument, into the concavity of the cap. Cases of inflammation and death of the nerve under this treatment had rarely occurred, and he had practiced it for several months past. He could not call to mind more than two or three unsuccessful operations. Lead cannot be placed in a cavity of sufficient thickness to prevent its bending under the force applied to secure a solid filling. He concurred with Dr. Harris, that no circumstance could warrant the filling of a tooth in the ordinary way, when the nerve is exposed or nearly so. Inflammation and death of the nerve, as well as intense suffering, must inevitably attend such an operation. He believed the nerve of a tooth could not be exposed so as to be touched in the slightest degree in excavating a cavity, even when the diseased portion was removed with the utmost care, without bleeding.

Dr. Westcott concurred with the gentleman in the last remark. The cavity of the nerve takes the form of the crown of the tooth, so that it comes to smaller points. In excavating a cavity you generally first ascertain that you have struck the nerve, by a small streak of blood from one of these needle-points of the cavity of the nerve. Whenever this happened in excavating, whether from necessity or accident, he considered it a great misfortune, almost certainly involving the ultimate destruction of the nerve.

Dr. Dwinelle.—I had formerly entertained the opinion that to expose the nerve-cavity was to warrant the destruction of the nerve. For the last two years and a half, my attention has been particularly directed towards restoring teeth whose nervous pulp had been exposed. I used sometimes to encounter the little oozing of blood which Dr. Westcott mentioned, and it gave me the same apprehensions. Whenever I have done so, I have considered the immediate or ultimate destruction of the nerve as certain. Of late, however, I have attempted to save the nerves whose membranes were broken, and have generally succeeded. About two years ago I was filling a tooth for a young lady, when I discovered that I had cut off a minute corner of the nerve. I immediately dipped a piece of cotton in a strong solution of camphor, placed it in the cavity and checked the blood. I filled the tooth carefully, watched it afterwards, and believed it did not die. She suffered no pain after the operation. I felt so interested in the operation that about six weeks ago I took out the filling, and found the cavity sound and healthy. I even went so far as to excavate around the point of the nerve, and found that there had actually been a deposit of bone over the exposed nerve. This will not appear incredible, when we reflect that in aged persons the nerve cavities are frequently filled with bone. With regard to Koecker's operation of using lead caps, I concur with Dr. Foster, that they may be so bent down by pressure in filling, as to come in contact with the nerve, and thereby become as objectionable and ruinous to the vitality of the tooth as would be the gold itself, except, perhaps, so far as lead may have the advantage of having the medicinal quality of being anti-inflammatory, gold being wholly inert. I have found the various preparations of lead very useful in subduing inflammation of the bone and nerves of the teeth.

Dr. Dwinelle suggested the application of some non-conductor under the cap, next to the nerve, to prevent cold or heat from affecting it—floss silk for instance.

Dr. Foster said that he had mentioned, in his preceding remarks, that he had applied Dr. Hill's composition, which was a non-conductor.

Dr. Dwinelle mentioned that Dr. Maynard had performed a like operation, applying beeswax under the cap.

Dr. Cleaveland.—With regard to using a non-conductor, I will state that I have used asbestos for nearly three years. It was first presented to my notice in the office of a gentleman of Havana. I had been with him about a month, when one day he was filling some teeth in which he used asbestos. He turned to me and asked me if I ever used the article. I said I had not, but now saw the utility of it. I have since used it in probably twenty cases, and cannot call to mind one single instance where I had to remove it.

Dr. Harris.—There is an article in the first volume of the American Journal of Dental science, by Dr. Solyman Brown, in which asbestos is recommended for this purpose.

Dr. Rich had tried the article, and it proved a failure.

Dr. C. O. Cone rose to present some remarks of Dr. Nelson on this subject, which were contained in his report on practical dentistry; the extract read, was to the effect that oiled silk at the bottom of the cavity was a sufficient non-conductor.

Dr. Rich.—Recommended gutta percha as a non-conductor. A thin layer to be placed between the bottom of the cavity and the filling. He said he had known many valuable teeth lose their vitality from the sudden transmission of caloric; he was confident that this might be prevented. He had lately used gutta percha for that purpose, with entire success. In several cases in which it was used, there was but a very thin partition of bone between the external cavity and the nerve. In his opinion, it was superior to asbestos, or any other substance which had been used for that purpose.

Dr. Harris suggested a solution of gutta percha in chloroform, the latter he thought would allay the irritation, and as it would evaporate immediately, a thin layer of the gutta percha would adhere firmly to the walls of the tooth; this was the suggestion of the moment, but he was of opinion it might be applied in this way with advantage.

Dr. Nelson stated that he had sometimes exposed the nerve without bleeding, but it was, he believed, owing to morbid action, the diseased bone being separated from the membrane.

Dr. Rich thought a great deal depended upon the delicacy of touch in the operation, whether hemorrhage took place or not.

Dr. Cleveland remarked, that since the introduction of chloroform he had made some experiments with it in excavating tender cavities. He thought to himself, if chloroform will destroy nervous sensation in the whole body, why will it not allay the irritation if applied to the tooth. The experiment was tried and with success, since which time he had resorted to chloroform, and with invariable success.

Dr. Harris corroborated the testimony of its utility by a number of experiments which he had made.

Dr. Rich stated that the discovery of chloroform had scarcely been published before an advertisement appeared in a New York paper, of a combination of chloroform and something else to prevent tooth-ache.

The society then adjourned, intending to resume the conversation on the following day, by taking up the "best method of applying arsenic."

*Third Day.*

Dr. Foster.—I did not experiment with arsenic until a long time after it was used; all that I heard respecting its effects influenced me against it. It was about two years after it became a general agent for the purpose of destroying nerves that I first tried it and according to the usual method. I applied it in a few cases, but the results were anything but satisfactory, and after a few trials I abandoned it. Soon after Dr. Maynard returned from Europe, meeting him one day in New York, and mentioning this subject, he remarked that he would when an opportunity offered, give me an insight into his peculiar method of using it. This information I never have obtained any farther than to know that he was using it in smaller proportions than others did. I commenced trying it upon this principle, in about the proportion of two grains of arsenic, to eight of morphine, and have generally succeeded in destroying the nerve, keeping it in ten or twelve hours without much pain in some cases, and without any in others. When pain has ensued, I think it attributable to the pressure of the substance used to keep the arsenic in the cavity, and that a cap placed over it would prevent suffering.

Dr. Westcott.—As I have heretofore given my views and particularly my objections in the Journal, in regard to the use of arsenic, for the purpose of destroying nerves, it is but justice to myself, to state that time and the information derived from others, who had been more successful in its employment, have somewhat modified the opinions set forth in the article to which I have alluded. This was written in the summer of 1845, and up to this time my own experience had been such as to elicit the opinions therein set forth. I need not here repeat the objections I then believed lay against its use.

I now only desire to say that I, at the present time, look upon the employment of this substance somewhat more favorably, or, in other words, I have to some extent, been enabled to overcome many of the objections then entertained, by adopting the mode of operating of one who had bestowed very great thought and attention to this very important branch of our profession—that of saving teeth after the nerve had been exposed. The practitioner to whom I allude is Dr. E. Maynard, of Washington City.

I had the pleasure in the winter of '46-'47 of not only examining many operations which he had previously performed, but in one case witnessing the performance, and I confess I obtained some new ideas upon this subject which have been of great value to me in my subsequent practice. For nearly two years previous to the time of writing the article alluded to, I had utterly refused to use arsenic in any case, but since comparing notes with Dr. Maynard, I have, though I must say very cautiously, used it in many cases, and in a majority of them with success so far as I now know. I described briefly his "modus operandi" in an article entitled "a Visit to Washington." and which was published in the March number of the Journal, 1847; and as most of the members present have doubtless seen that article, I need not describe it here.

About a year and a half has elapsed since, during which time I have continued to experiment with this substance, yet at no time with as satisfactory results as I could wish. If the only object was to avoid, or to diminish the pain of destroying nerves, then arsenic deserves the highest eulogy, as my own experience proves; pain scarce ever need be inflicted in destroying a nerve. But in reference to the ultimate result, the health and safety of the tooth, it has been by no means as certain, as destroying the nerve at once with an instrument. However humiliating it may be, I am bound to say that in my hands quite a large proportion of these operations, where arsenic was employed, have resulted in the formation of alveolar abscesses, and I think the proporion is at least three to one, as compared to the unfavorable cases, where the nerve was removed by an instrument. It is indeed a very rare occurrence to have abscess result from removing a healthy nerve with an instrument. It is frequently the misfortune of the dentist to be only able to choose between *evils*, and fortunate is he who always selects the least. In this light, and in this light alone, can we view the operation of destroying nerves by whatever method it is effected, for at best it so far weakens the vitality of the tooth as to render it *in all cases liable* to prove uncongenial to the surrounding parts. But even admitting this contingency to pertain to every case, yet it often is true that the destruction of the nerve, is by far a less evil than extraction, and still less than would result by leaving the tooth with an exposed nerve.

Dr. Harris,—Did not know that he could throw any additional light on this subject. Soon after Dr. Spooner of New York published to the world his application of arsenic to exposed dental nerves, he was led to make some experiments with it, which, however did not induce him to continue the practice very long. But about the year 1839, he began to repeat these experiments, and from that time to 1842 or 1843 had applied it, in perhaps three hundred cases, for destroying the vitality of the pulp previously to removing it, in order to fill the cavity of the tooth. During this time he had an opportunity of examining the results of cases in which arsenic had been used by other practitioners in Baltimore, and other parts of the United States. In at least three out of four cases, alveolar abscesses resulted within from three months to two years. He was again induced to abandon the use of this agent, believing that it rendered the teeth obnoxious to the surrounding parts. After Dr. Westcott's return from Washington, hearing his description of the elegant manner of operating by Dr. Maynard, he was again induced to repeat the operations. Since that time he had applied arsenic to about twenty teeth, in the mouths of persons whom he felt sure of seeing again, and of ascertaining the result, and he must say that he had been more successful than he anticipated. He had also filled some eight or ten teeth in which the nervous pulp had previously been destroyed by suppuration, after having brought about a healthy action in the parts at the extremity of the roots from which a morbid fluid was secreted. He had thus far succeeded in a majority of these cases. In one case, that of a lady from South Carolina, he filled the pulp cavities and roots of the upper cuspidati. These teeth had previously given rise to alveolar abscesses. The roots were carefully cleansed and injected with a solution of chloride of soda for three or four successive days when they were filled. Two weeks after a small abscess or gum-boil, formed over each, which soon broke and discharged. Although the presence of the teeth are productive of a small degree of irritation, they are less hurtful and more useful than artificial teeth. In some three of the other unsuccessful cases, there have been similar small abscesses, but the morbid effects attending them were not sufficient to cause any manifest inconvenience. A few days days before he left home, he examined a molar filled by Dr. Cone. For some two or three weeks after the operation the patient had suffered a slight degree of irritation, but it has not subsequently occasioned inconvenience. My manner of applying arsenic said Dr. H. is simply this; I take one grain of arsenic and one of Sulphate of morphine, mix them together and divide into thirty powders, I then moisten a small particle of cotton with water, sometimes with creosote, and sometimes with laudanum and absorb as much of this minute powder as I can upon the cotton. Having previously exposed the nerve, I put this in contact with it, and then seal up the cavity with wax. I rarely permit it to remain more than seven hours.

Dr. Dwinelle.—When I commenced the practice of my profession, for what I then considered good and substantial reasons, I was opposed to the use of arsenic. My prejudice arose from the fact of an acquaintance of mine having been killed by a quack, in treating a cancer with arsenic; also from the fact that several of my friends were laboring under disease of the jaw and alveolar abscesses, occasioned by arsenic having been applied to their teeth. I ultimately found that a substance more powerful than creosote was desirable, if safe, and I commenced experimenting with arsenic. I used it with caution, in minute quantities, sealing up the cavities with wax and cotton. I was not always successful. At times its use was wholly unaccompanied with pain, and with the best results; experience rendered me more perfect and successful, but I was never decidedly confirmed in the use of it, till I had a conversation with Dr. Maynard on the subject. My experiments with this article, since that time, have been almost invariably successful. When it has produced alveolar abscess, they were but slight making usually but one discharge, and then healing up permanently. But after all I prefer the method of destroying nerves with instruments. It is my conviction that in most cases an alveolar abscess is less likely to ensue when you take the nerve out bodily at once, and fill the internal cavity with gold, than when you give it the more protracted treatment with arsenic. I finally made up my mind seldom to use arsenic, and then in the manner described. I never mix creosote and morphine in any relative proportions. I do not let it remain more than twenty-four hours at a time, being afraid of its poisonous influence extending beyond the nerve.

Dr. Dunning asked if in his opinion, the absorbing powers of the nerve continued after it was dead, so that the arsenic could be taken up?

Dr. Dwinelle.—The nerve is impregnated with arsenic, and when it is dead, I think the arsenic, if left to itself will produce manifest disorganizing effects at the extremity of the tooth.

I know a lady, one of whose superior molar teeth, below the crown is completely riddled by arsenic. The carious cavity was filled with arsenic, and renewed after the nerve was killed from day to day, to cure the *soreness* ; and finally the dentist (?) in his despair, filled the cavity with arsenic, and completed the operation with tin foil. The root of the tooth is riddled like a honey-comb.

Dr. Cleveland.—In the plunging system, supposing a molar tooth decayed upon the posterior portion, while the crown is solid above, and you wish to destroy the nerve around the fang, how do you reach the whole extent of the cavity of the root?

Dr. Dwinelle.—I use Dr. Maynard's instrument of untempered steel, filed down to the fineness of a horse-hair. This instrument can be bent into any shape, and you have only to crook it over the arch or border of the cavity, The nerve is, I believe less sensitive as you approach the point of the fang. Dr. Dwinelle here described an instrument invented by Dr. Maynard, for the purpose of removing particles of extraneous matter from within the tooth. It was prepared with a succession of barbs at the point and sides, like a barley beard in order to draw out foreign substances. When I have exposed the nerve in removing the crown of a tooth, for instance I make it a point to seize upon that moment to destroy it for the purpose of gaining ingress into the nerve without much pain, as the concussion of the operation benumbs the nerve for the moment. I apply a very little concentrated creosote, which enables me to enlarge the orifice sufficiently to get an instrument into the cavity. If practicable, I enlarge the cavity with a conical or V shaped burr, and then plunge in the instrument and destroy the nerve. With regard to their being a small alveolar gathering afterwards, I quote Dr. Maynard as authority, who says he finds one of these occasionally after an operation, but that it never occurs a second time.

It was here suggested that the Society should listen to Dr. Hill's description of his composition for fillings; but inasmuch as the materials composing it were not to be made known, any remarks from Dr. Hill on that subject were deemed not in order.

The next part of the subject proposed for discussion, was the best manner and material for polishing the bone.

Dr. Dunning was in the habit of polishing with prepared pumice stone, using a stick of red cedar—an ordinary pen-holder. In reducing the inequalities on the surface of the stopping, he frequently used the stone of Ayre, which is obtained in the hardware stores.

Dr. Westcott had used the Arkansas oil-stone. The only objection to it was that it was very apt to get cloggy; if that could be obviated it would be the best thing possible. He took pains to get it in thin strips, so as to use between the front teeth. It gives a fine polish, but tires one's patience in using. The Scotch stone would be quite as good if it could be obtained in as thin slips.

Dr. Cone had found an article known as leather wood, which is procured in the south, and is very porous. This being first put into warm water, and then dipped into a solution of glue or size, with flour of emery of various degrees of fineness, make a beautiful material for polishing. I have sometimes used even rotten-stone in this way. In completing the polishing operation, I use floss silk. In this I may appear more nice than wise. I wish here to make an inquiry. In the microscopic examination of the teeth, we know that decay commences first on the inner side of the tubes. Now the question is this: is the unequal surface, by the retention of extraneous matter, the sole cause of its more rapid decay? It is undoubtedly an important cause, but is it the whole cause? And must not these open tubes (microscopically speaking,) though polished as fine as possible, be left open for the action of foreign matter? And if not, how are these partially closed by the finer material used, which we know to be the fact?

Dr. Harris.—After the file scratches have been to a considerable extent removed by the polishing material, the subsequent process employed in smoothing the surface has a tendency to close up the mouths of the tubules, or rather of the cellules for I am inclined to believe the doctrine of the tubularity of dentine erroneous. For example, I find this hollow cylinder of my pencil too large to retain the part which slides into it. By simply rubbing its orifice with a burnisher, I close it sufficiently to retain the other part; just so the mouths of the open cells of the teeth are, at least to some extent, closed.

With regard to the various materials used for polishing, I have employed Arkansas oil-stone prepared in thin slips, but the difficulty mentioned by Dr. Westcott is a very serious one, for in the course of a year, one would break and destroy perhaps fifty dollars worth. A few months ago I had some reduced to a very fine powder, which I have been in the habit of using on thin slips of wood, and on floss silk moistened with water. This is far more economical; a thimble-full lasting a long time. It requires a vast amount of labor, however, to reduce it to a sufficiently fine powder, and can only be done on a slab or in a mortar. It might, perhaps, be done by heating to a red heat and then plunging it in water, but I am disposed to think the sharp angles of the particles would be injured by such treatment.

Dr. Dwinelle.—Inasmuch as my predecessor has led off into the field of theory, permit me to follow in his footsteps, and speak of the osseous deposit mentioned by Dr. Cone. He seems to assume that when the surface of the bone is polished, the diameter of the tubuli is not as great as when broken or uneven. Suppose we take a piece of enamel, which, in fact, is like a honey-comb, with the angles broken and uneven. When it is filed, it presents a succession of acute angles—a velvet surface such as we give to a gold stopping when we want it to assimilate in tone to the surface of the tooth. We now use a finer file, and we leave an undulating surface, the top of the acute angles being cut off. We next use pumice stone, and it is not *critically* speaking, reduced to a plane yet, there is now a succession of cylinders, like the Giant's Causeway, microscopically speaking. So we proceed till we give a complete polish. Now, I ask, have we not reduced the real given surface? Examine that polished surface, and you will find the diameter of the tubuli just as broad as before? but you have reduced the actual surface and overcome the capillary attraction of its broken tubuli. To show the delicacy of this attraction, let us take to the process of lithography. Take a broad lithographic polished stone; you execute the drawing with a crayon composed of wax, tallow and lampblack; you pour on sulphuric acid, which etches it all but where the wax lies; you now take a wet sponge and rub over the stone, and the capillary attraction causes the water to be retained sufficiently on the etched surface to repel the ink from the roller. This shows the force of capillary attraction on a rough craggy surface. In the rough filed surface of the tooth bone, the fluids and food are retained by capillary attraction. With regard to the question at issue, I have used but few substances for polishing teeth. Ayrshire stone I can generally whittle down for the occasion, but it soon wears away. Pulverized pumice is excellent to precede it. I have not used Arkansas oil-stone much, on account of its clogging; oiling it will carry off the substance accumulated, but this makes too dirty an operation. I have long used another very convenient article—a little file used by jewellers. I have seen the same on Dr. Parmly's and Dr. Dunning's tables; it is very fine and flat.

Dr. Dwinelle, at the request of several members, now proceeded to describe his process of bleaching dead teeth.

Dr. D. prefixed his remarks by saying—Who of us have not had our better feelings and our conceptions of beauty and harmony shocked, at seeing a dead black tooth in a dental row of living and sparkling ones, like a jet in a setting of pearls. At first sight it often strikes one as a *deficiency* altogether, but on a second, we find the foul spot has a fadeless and permanent hue, enough to excite the envy of a Chinese beauty.

Although these instances are comparatively rare, yet they are sufficiently frequent to keep our indignation and regret in a decided state of health and activity—indignation at the blundering operator, or regret for the accident which caused the "dark transaction!"

These deformities are often of such decided and unpleasant character, that the unfortunate subjects of them often seek relief in having the dead member dislodged, and an artificial one placed in its stead. Up to within a few years past, I have occasionally in some extreme cases, yielded to the solicitations of my patients in this respect, but ever with unpleasant reflections and doubts exceedingly annoying. I have ever been impressed with the idea, that the sacrifice was too great, even for beauty's sake; for oftentimes, even though the tooth is of ebon shade, it is comparatively sound and as firmly set in the alveolus as its pale-faced neighbors.

Abscesses and fistulous openings are often associated with the kind of teeth under consideration; in attempting to cure these, we accidentally discovered a method of restoring the teeth to their natural whiteness and beauty.

Premising that all present know that the teeth are of such porous texture and quality that they are capable of being injected, and that the discoloration of which we speak, is occasioned by the dead blackened nerve in a semi-fluid state, being disseminated throughout the bony structure of the tooth. I will proceed briefly to recount my remedy.

To illustrate, I will refer to one or two of my earliest cases. Mrs. ——, a lady, residing in Cazenovia, had a superior frontal incisor plugged with gold by an unskillful operator, about ten years ago. The operation was exceedingly painful, according to the representation of my patient, the filling at the time being applied directly to the exposed nerve.

She suffered much for a number of weeks, and soon after the pain subsided, a large protuberance was raised in front which pressed out the lip and even gave the nose a permanent elevation, as in the case of a swelled face, very much disfiguring her countenances. While I was operating to discharge the reservoir of matter which caused the protuberance, I suggested to her that I would like to see what I could do towards bleaching this tooth as well as to cure the abscess and reduce the protuberance. She gave me permission to do so, and I proceeded to drill through the sound part of the tooth, leaving the lateral fillings as they were. I drilled into the nerve cavity, and with a hoe-shaped instrument I drew out a quantity of pastry substance, something like ink; I then washed out the cavity with a solution of camphor, and filled the root with cotton from the apex down to the pulp cavity of the tooth; I then cleaned it out as much as possible, and for experiment introduced cotton of various colors, to see how much the tooth was whitened. The yellow cotton produced the most natural color, yet there was a dark, dead medium. I then filled up the cavity with a solution of lime and sealed it with white wax. She came next day. I took out the lime water, found it was considerably stained; but the tooth looked better. I removed some more of the internal surface by scraping, and continued the application of lime till I thought something else was required. I next introduced soda by way of change, alternating too with chloride of lime. After having completed this process, I filled the tooth to the extremity with gold, using a very fine instrument, filling every point, water and air tight. I then closed up the cavity and went to work at the outer surface of the tooth, taking off the surface, which was even darker than on the inside. I at length succeeded in raising the color of that tooth to as high a tone if not higher than its neighbor at its side. In the course of about two months, however, it subsided a shade darker; but it is now as much whiter than before, as gray is whiter than charcoal. This is one of my comparatively unsuccessful operations; I

had another case where the tooth was as black as this, which I challenge any one to distinguish from others around it. There are cases where I cannot succeed as well as I could wish. I operated on the teeth of two sisters in one family, and succeeded beyond my expectation. I brought the teeth up from a dark snuff color to a rather rich golden white. They were very much gratified, and consider the operation entirely successful, which I do not.

Previous to my attempting to bleach teeth, I had not heard of the operation, and know that I was the first to practice it in my vicinity.

It has been my good fortune to restore a large majority of the stained teeth which have passed through my hands, to their original color and beauty. I regard it as an operation which, in proper hands, promises much, and I feel sanguine when I make the declaration, that a little perseverance, aided by a common share of skill, will greatly improve, if not completely restore four cases out of five. Should my friends attempt the operation, I trust they will persevere, and not predicate the success which surely lies before them upon one or two of their first failures.

Dr. Dunning inquired if he had tried it where amalgam had discolored the teeth.

Dr. Dwinelle said he had not.

He then inquired if it would not be well to cut a great deal from within the tooth?

Dr. Dwinelle said he did so. When I have finished bleaching the tooth, I try the effect of gold foil on the inside to see to what pitch I have brought it. I had almost forgot to mention in regard to the first case that I cut out the protuberance, healed up the sinus, and the lady's nose has assumed its natural character.

Dr. Harris said he had tested the bleaching qualities of chloride of lime and soda, and always kept a bottle of one of them containing a solution for this purpose.

Dr. Foster related a case of a lady who had the misfortune to have a dead front incisor so long neglected that its blackness almost deterred him from the hope that it could be restored in strength or color. All the other teeth in the mouth were perfect in number, and of that beautiful pearly white texture, so rarely seen. After a long and very laborious process of excavation, the blackness was entirely removed, and the tooth filled into the nerve cavity. He then found the whole front surface of the crown of the tooth so thin that unless some intervening substance could be placed between the gold and the enamel so as to restore the color of the tooth, his labor would be in vain, for it would be a gold tooth in color as well as weight; upon placing a small piece of a newspaper under the front surface from curiosity he could readily distinguish the letters through the enamel. This suggested the experiment of trying other paper, and a piece of blue letter paper, cut to the form of the thin surface of the enamel, but not allowed to extend quite to the edges of the cavity, was found to answer the purpose, and when the gold was pushed carefully under it and the operation completed, the difference in point of color was hardly observable. This operation was performed the week previous to this meeting, and its ultimate utility undetermined, but the chances of an unfavorable termination, cannot, he thinks, be increased by the use of the paper, provided it does not extend to the edges of the cavity, and the filling is so firm and compact, as that moisture shall not reach it.

Dr. Rich had tried the same experiment, only he had used tissue paper, which was not so good.

Dr. Westcott had lately treated a tooth which was perfectly dead and extremely discolored. He drilled into the tooth, wound the apex of the syringe with cotton, to prevent the fluid from running back, and thus forced the fluid through the apex of the tooth. He did not like to trust the other method of cleaning, as foul matter was liable to be left at the end of the root.

With regard to the substance used for bleaching, he used either chloride of lime or chloride of soda, taking the precaution to free it from all acid, before it was employed. So far as his experience could decide, it was an operation promising very little. Treated one tooth four or five weeks with but slight change of color.

The Society adjourned till eight o'clock in the evening.

*Third Day*, 8 *o'clock*, *P. M.*

The part of the subject next taken up, was the best means of keeping the cavity dry for filling.

Dr. Rich.—Some years ago, as an experiment, I dried a cavity with a piece of newspaper. It answered the purpose so far as to make the cavity dry, but some particles of the paper were left adhering to the walls of the cavity. Subsequently I tried different kinds of paper, and found tissue paper slightly sized, to answer the best for this purpose. I prepare it by rubbing until it is quite soft, and then tear it in strips of an eighth, quarter and a half an inch wide, and three or four inches long, so that I have three sizes. Each strip is then twisted into the form of a rope, and it is ready for use. When I wish to dry a cavity I take a strip of twisted paper and pack it into the cavity until there is enough in to absorb all moisture leaving the end projecting. To remove the paper twist the projecting end hard, and the paper will come out easily leaving the cavity dry. I never was able to make a cavity perfectly dry with cotton, but I can with paper. A simple experiment will illustrate the superiority of paper, prepared as I have directed, over cotton, for absorbing moisture; place two drops of water upon a piece of glass, and attempt to take one up with a lock of cotton; you will find it difficult to do so. Touch the other drop with a piece of prepared paper, or you may use a piece of newspaper, it will absorb all the water, and if the paper be pressed upon the glass, it will leave the glass perfectly dry.

I often use paper with great advantage, when I wish to fill cavities that may be near or partly under the gum. In such cases, the paper is twisted so as to form a small cord and wound around the neck of the tooth, it is then pressed up under the edge of the gum, and the ends secured with a piece of thread, this will prevent any saliva from entering the cavity.

Where there is an extraordinary flow of saliva, I use paper to absorb it, particularly when it is necessary the mouth should remain open a long time. For this purpose I use thick blotting or filtering paper, it is made up into rolls about the thickness of a finger, say from two to three inches in circumferance, and from three to four inches long, according to the size of the mouth. Have ready a number of these rolls and when you wish to use them, direct the patient to raise the point of the tongue to the roof of the mouth, bind the roll of paper so that it wil lay inside of the arch of the teeth, and when you have placed it there, the tongue must be gently pressed down upon it to keep it in place. The paper will absorb a large quantity of saliva. When the roll becomes saturated it can be changed with great facility. If it becomes necessary to stop the flow of saliva from the salivary ducts, the thick blotting paper may be used for that purpose, by folding three or four thicknesses of it together, and placing it between the cheek and gum, so that it will cover and press upon the mouth of the duct. The surrounding parts must be made perfectly dry and the paper applied quickly; if this be not done dexterously, so that the paper and membrane are dry where they come in contact, they will adhere so closely, that you will have to wet the paper before it can be removed with safety. If you attempt to remove the paper while it remains dry, a portion of the mucous membrane will adhere to it.

I have been thus particular in describing the manner in which I use paper, as I believe it will be new and interesting to many. When I was experimenting with it which is some years since, I mentioned the result of my experiments to Drs. Cleaveland, C. A. Harris, Solyman Brown, E. Maynard, E. Noyes, L. Roper, E. Townsend, A. Westcott, W. H. Dwinelle, E. J. Dunning, C. C. Allen, J. H. Foster, and others, and it was a new idea to them at that time.

Dr. Foster said, that Dr. Rich had entered so carefully into the subject, and his method of drying cavities and preventing the flow of saliva being very similar, he had but a few remarks to make upon this question. For drying the cavities, he used the finest old linen handkerchief, well worn, that could be had; these, his lady patients, many of them, seeing the use to which they were applied, were in the habit of furnishing him with abundantly, for which he was much indebted. He used linen doubled, for absorbing the saliva in filling the under teeth, placing one of these in the requisite position, and retaining it there by means of an instrument made for that purpose, the handle of which the patient holds with the left hand. Sometimes he has found it necessary to change these doilies three or four times in filling one cavity to prevent the flow of saliva into it. It is important that these doilies should be pure linen, and he always depended on the judgment of the ladies, and not his own, in procuring them. In filling the upper teeth, no other precaution is necessary to keep the cavity dry, than to hold a small fold of one end of a doily with the fingers of the left hand against the inside of the tooth, and with the other end occasionally to wipe away the saliva as it oozes out from under the upper lip around the alveolar ridge in front.

Dr. Cleveland inquired what method he took to relieve the moisture in the event of its getting in before he completed the filling?

Dr. Foster said he took out the gold and tried again, except in a very few cases he could succeed in keeping the cavity dry.

Dr. Harris said he could show a tooth filled without drying, which, at the time he had no expectation of preserving more than a year or two, in consequence of the frail texture of the organ. It is now as perfect as when first filled without drying, which, at the time, he had no expectation of preserving more than a year or two, in consequence of the frail texture of the organ. It is now as perfect as when first filled. He felt the importance of keeping the cavity dry, but had met with cases where he could not do it. In such cases, he expected by pressure to force every particle of moisture from the cavity.

Drs. Wescott and Rich thought it could not be done.

Dr. Cleveland was in the habit of using unsized fine cotton cambric for preventing the moisture from getting into the cavity. He tore it in strips about the eighth of an inch wide, or more, as occasion required, and a quarter of a yard in length. It enabled him to absorb the moisture quicker than anything else.

Dr. Harris.—I always aim to keep the cavity dry, but sometimes I must confess, I cannot. I have sometimes removed the plug half a dozen times, until I have been compelled to introduce the gold when the cavity was wet. I have a filling in one of my teeth, introduced by a student of mine, in which he failed to keep the cavity dry, and I venture to say it will be as perfect at the end of ten years as now. With regard to the best form to give to gold previously to introducing it in the cavity, the method I pursue is to make a narrow fold or roll from a piece of foil, varying in width from a quarter of an inch to an inch.

The chairman said that he sometimes made a ball as hard as he could possibly roll it.

Dr. Cleveland had quite a different method of preparing the foil. He took a sheet of foil, and cut a strip entirely around, till the whole sheet was cut up. For an ordinary cavity, he took a strip half a yard in length. He placed this upon a velvet cushion, and with a blunt condenser broke up the smoothness of the strip of gold. He then formed it into a perfect string, having something the appearance of a gold chain. The advantage of this was, that the parts interlock so as to form a perfect ball. The doctor here exhibited a string of gold thus prepared, and showed his method of preparing it much to the gratification of the Society. He said it was first suggested to him by his brother Thomas. With regard to checking the flow of saliva, he had never used any of the articles mentioned. He was in the habit of using his finger and thumb for that purpose.

Dr. Westcott gave his views at length upon the subject of keeping cavities dry, describing the means employed, and demonstrating the necessity of the greatest pains to exclude all moisture during the operation of plugging.

[Dr. Westcott having since this meeting, made some improvements in the means of freeing the mouth from saliva during the process of filling, has decided to embody his remarks upon that occasion in a separate article upon this subject; they are therefore omitted here.]

Dr. Rich desired the opinion of the members, whether it was not considered important, to have the surface of the filling continuous with that of the tooth? The general opinion was that it was better to have it so, in order to have a perfect joint; also to have a depression in the center of the filling.

Dr. Rich said he had adopted a plan to prevent the flow of moisture from the palate, which was to make the patient draw in his breath through the mouth, and expel it through the nostrils.

Dr. Morton, of Boston, here described an apparatus which he had invented to obviate the flow of saliva. The apparatus cannot be described in words, without a drawing. It is sufficient to say that it resembles a miniature fife, and is attached to the under jaw, to collect the saliva.

Dr. Dwinelle. I have considered this subject of such importance, that whenever I have failed in keeping a cavity dry, I have kept mighty dark. Submarine operations may do for ships, but not for dentistry. In order to obviate the flow of saliva, I have resorted to curious contrivances. One of them is this: in filling an inferior molar on its grinding surface, I have taken a fine slab wet it on either side, and dipped it in white wax thereby making sheets of wax, You can have them as thin or as thick as you please. I wipe my tooth dry with Dr. Rich's paper, after which I build a coffer dam around the tooth to be filled, and proceed. Sometimes, when the flow of saliva is very abundant, I take a small syringe, wind a piece of copper wire around the middle of it, so as to leave a ring in which to insert my finger, and with my thumb in the ring of the piston, I pump out the mouth with my left hand. In this manner I have filled an ordinary tumbler with saliva, during a single operation. I agree with Dr. Westcott that moisture will prevent the gold from cohering. When there is a probability of the gold having absorbed moisture, I anneal it anew. You will sometimes find the first leaves of foil work admirably, but after you get to the back part of the book it is a different article. I take these leaves and hold them over the blaze of a spirit lamp, (not of pure alcohol, for that give too hot a flame,) and it is astonishing how the surface of the gold will adhere. Care should be taken to keep gold foil dry, by keeping it in a drawer by itself, with a weight upon it covering the whole surface of the book.

The evening being far spent, the Society preceeded to transact the little remaining business, and adjourn till the next year. The members had conducted the three days discussion in an excellent spirit, and were all highly gratified with the mutual interchange of each other's views and methods of practice.

